# Clinical Improvement With Transcatheter Edge-to-Edge Tricuspid Repair Irrespective of Right Atrial Pressure

**DOI:** 10.1016/j.jacadv.2024.100862

**Published:** 2024-02-27

**Authors:** Lina Ya'Qoub, Hope Caughron, Atif Qasim, Kirsten Tolstrop, Francisca N. Delling, Clifton Watt, Tom Nguyen, Tobias Deuse, Ramin E. Beygui, Vaikom S. Mahadevan

**Affiliations:** a University of California-San Francisco, San Francisco, California, USA; b University of Massachusetts Chan School of Medicine, Worcester, Massachusetts, USA

Severe tricuspid regurgitation (TR) refractory to medical therapy remains challenging to manage at the present time. Transcatheter therapies developed in recent years are being performed as part of investigational studies.^1^, ^2^, ^3^ Limited data exist on the effect of hemodynamics, specifically the baseline right atrial (RA) pressure, on outcomes of transcatheter edge-to-edge repair (TEER) of the tricuspid valve.^4^^,^^5^ We aim to assess the effect of baseline RA pressure on the outcomes of patients with isolated severe TR who underwent TEER of the tricuspid valve as a stand-alone procedure using the MitraClip system.

We included patients who underwent TEER for TR in our medical center from October 2019 till August 2022. Patients were divided into 2 groups: those with a baseline RA pressure of <10 mm Hg and ≥10 mm Hg. The study had approval by the Institutional Review Board and clinical data were collected from the electronic medical records retrospectively. The primary outcome was NYHA functional class and Kansas City Cardiomyopathy Questionnaire (KCCQ) score post-TEER. Secondary outcomes were in-hospital and 30-day all-cause mortality, residual TR ≥ moderate, right ventricular dysfunction ≥ moderate, and heart failure hospitalization. All descriptive analyses were conducted using means for continuous variables and frequencies for categorical variables. Comparison between the groups was performed using the 2-sample *t*-test and Fisher exact test. All analyses were considered statistically significant if the *P* value was <0.05.

A total of 27 patients (59.2% were females) were included in our registry, with a mean age of 75.6 years old. In this cohort, 10 out of 27 patients (37%) had a permanent pacemaker lead traversing the tricuspid valve, 14 out of 27 (51.8%) patients had previous cardiac surgery, while 23 out of 27 patients (85.2%) had atrial fibrillation. Prevalence of renal failure was observed at baseline in 11 out of 27 patients (40.7%) and coronary artery disease in 14 patients (51.8%). Patients had severe TR in 63% and torrential TR in 37% of cases, with mean effective regurgitant orifice area of 48.6 mm^2^, vena contract of 8.2 mm, and regurgitant volume was 65.7 ml at baseline. The clip was successfully deployed in 24 out of 27 patients (88.9%), while in 3 out of 27 patients, the clip could not be deployed due to inadequate visualization of leaflets or presence of commissural regurgitation. We performed TEER using XT (4/24, 16.7%), XTR (5/24, 20.8%), and XTW (15/24, 62.5%). Independent leaflet grasping technique was performed in 7 out of 27 patients (25.9%). The fluoroscopy time of the procedure ranged from 9.7 minutes to 64 minutes.

NYHA functional class was III (22.2%) or IV (77.8%) at baseline. Post-clip, NYHA functional class I and II was noted in 91.6% and the mean KCCQ score improved from 35.6 to 49.9 (*P* = 0.0002). Post-clip, TR of less than severe was noted in 91.6% of patients, resulting in TR reduction ≥1 grade in 91.6%. There was no procedural or 30-day mortality. Early single leaflet detachment occurred in 3 out of 24 patients (12.5%) before discharge. Time to discharge ranged from 1 day to 13 days, with a median of 1 day. Tricuspid valve surgery was performed in 1 patient (4.2%). This was the patient who had late single leaflet detachment with severe residual TR within 30 days of the procedure. All-cause mortality occurred in 5 patients (20.8%) at a mean follow-up of 15 ± 4.6 months; 2 patients had progressive heart failure symptoms and were transitioned to hospice/palliative care after 2 years post-TEER, 1 patient died of cancer, 1 patient died of gastrointestinal bleeding, and the last patient died of severe respiratory failure secondary to coronavirus infection. On subanalysis of the cohort based on RA pressure (<10 mm Hg, n = 13; ≥10 mm Hg, n = 11), there was no difference in baseline characteristics (including age, male, hypertension, diabetes mellitus, atrial fibrillation, right ventricular dysfunction, pacemaker, and previous cardiac surgery) between the 2 groups (*P* ≥ 0.36), except for renal failure (defined as Cr >1.3 mg/dl), which was significantly more common in patients with RA ≥10 mm Hg (15.4% vs 54.5%, *P* = 0.04). There was no difference in the number of clips implanted (1.23 vs 1.45, *P* = 0.07), leaflet detachment complications (23.1% vs 0 *P* = 0.09), KCCQ score at follow-up (49.8 vs 50.1, *P* = 0.40), NYHA functional class at follow-up (NYHA functional class I or II 92.3% vs 100%, *P* = 1.00), in-hospital mortality (0 vs 0), length of hospital stay (2.2 ± 3.3 days vs 2.8 ± 3.2, *P* = 0.80), and 30-day mortality (0 vs 0).

**Figure 1:**
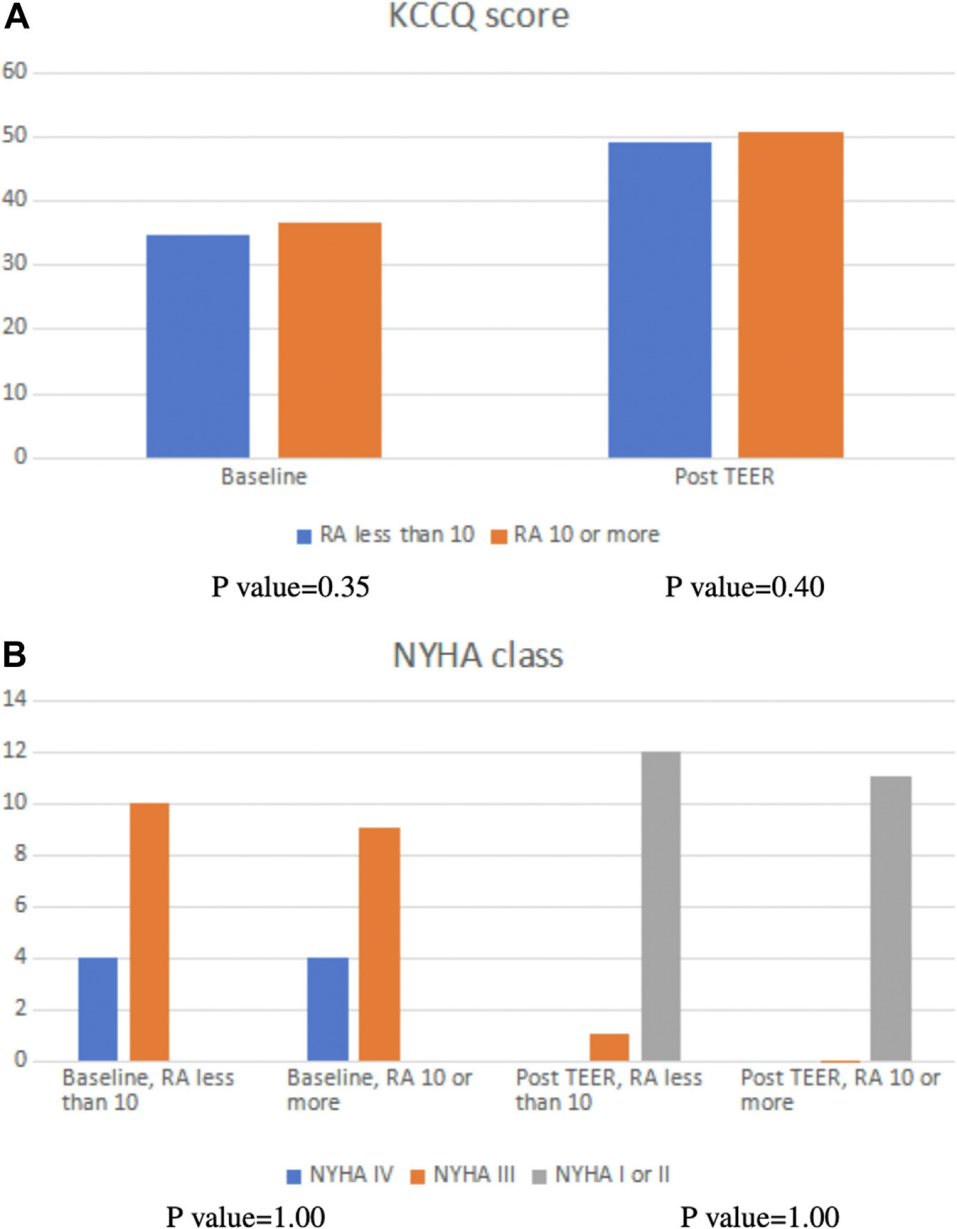
KCCQ Scores and NYHA Class Based on Baseline RA Pressures, Pre- and Post-Intervention (A) KCCQ score stratified by RA pressure at baseline and post-TEER. (B) NYHA functional class stratified by RA pressure at baseline and post-TEER. KCCQ = Kansas City Cardiomyopathy Questionnaire; RA = right atrial; TEER = transcatheter edge-to-edge repair.

Our study demonstrates that TEER in patients with isolated severe TR using the MitraClip device as an off-label procedure was associated with clinical improvement illustrated by NYHA functional class and mean KCCQ score on follow-up irrespective of baseline RA pressure (Figure 1). Technical success rate (88.9%) in our cohort is consistent with prior reports, despite including patients who would have been otherwise excluded by other registries, including patients with permanent pacemakers.^1^, ^2^, ^3^, ^4^ While there are studies showing the impact of pulmonary hypertension in patients undergoing TEER for TR, there are limited data on the impact of baseline RA pressure on the outcomes of patients undergoing TEER for isolated TR.^4^^,^^5^ Our study is theory-provoking and high-quality data from randomized clinical trials are needed to confirm these findings.
